# Evolution patterns of probable REM sleep behavior disorder predicts Parkinson’s disease progression

**DOI:** 10.1038/s41531-022-00303-0

**Published:** 2022-04-05

**Authors:** Guanyu Ye, Xiaomeng Xu, Liche Zhou, Aonan Zhao, Lin Zhu, Jun Liu

**Affiliations:** 1 Department of Neurology and Institute of Neurology, Ruijin Hospital, Shanghai Jiao Tong University School of Medicine, Shanghai, China; 2 CAS Center for Excellence in Brain Science and Intelligence Technology, Ruijin Hospital, Shanghai Jiao Tong University School of Medicine, Shanghai, China

**Keywords:** Parkinson's disease, Parkinson's disease, Parkinson's disease

## Abstract

The course of REM sleep behavior disorder (RBD) variates in the early stage of Parkinson’s disease. We aimed to delineate the association between the evolution pattern of probable RBD (pRBD) and the progression of Parkinson’s disease (PD). 281 de novo PD patients from the Parkinson’s Progression Markers Initiative database were included. Patients were followed up for a mean of 6.8 years and were classified into different groups according to the evolution patterns of pRBD. Disease progression was compared among groups using survival analysis, where the endpoint was defined as progression to Hoehn-Yahr stage 3 or higher for motor progression and progression to mild cognitive impairment for cognitive decline. At the 4th year of follow-up, four types of pRBD evolution patterns were identified: (1) non-RBD-stable (55.5%): patients persistently free of pRBD; (2) late-RBD (12.1%): patients developed pRBD during follow-up; (3) RBD-stable (24.9%): patients showed persistent pRBD, and (4) RBD-reversion (7.5%): patients showed pRBD at baseline which disappeared during follow-up. The RBD-reversion type showed the fastest motor progression while the RBD-stable type showed the fastest cognitive decline. At baseline, the RBD-reversion type showed the most severe gray matter atrophy in the middle frontal gyrus, while the RBD-stable type showed gray matter atrophy mainly in the para-hippocampal gyrus. Four types of early pRBD evolution patterns featured different brain lesions and predicted different courses of motor and cognitive decline in PD.

## Introduction

REM sleep behavior disorder (RBD) is a parasomnia characterized by dream enactment behavior (DEB) and REM sleep without atonia (RWA)^1,2^. RBD is frequently observed in Parkinson’s disease (PD), with a prevalence of 16–47%, and can predate or follow the onset of motor symptoms^3^. Mounting evidence suggests that PD patients with RBD (PD-RBD) show distinctive clinical features compared to PD patients without RBD^4^, including more severe motor disability, greater cognitive impairment, and faster disease progression^5^, which indicates that PD-RBD may represent a distinct phenotype.

However, longitudinal data have shown that RBD symptoms do not always remain stable in PD patients. Several studies found that patients with de novo PD had an elevated overall rate of RBD during follow-up^6,7^, indicating that PD patients without RBD symptoms at onset might develop RBD over the course of the disease. Meanwhile, decrease^8^ and/or disappearance^9–11^ of previously existing RBD symptoms over PD progression were also identified in accumulating publications, reporting RBD remission rates of 12.0^11^ to 33.3%^9^. The heterogeneity in the evolution pattern of RBD symptoms may reflect the difference in stage and affected brain regions in PD patients. Therefore, this evolution pattern may have a prognostic value. It will be useful in clinical practice to cluster PD patients by the evolution pattern of RBD symptoms and to explore its relation to PD disease progression.

In the present study, we aimed to identify the variation of probable RBD (pRBD) over PD course, to explore its relation to motor and cognitive progression in de novo PD patients, and to elucidate the potential underlying mechanisms using neuroimaging approaches.

## Results

### pRBD evolution pattern in PD patients

**Fig. 1:**
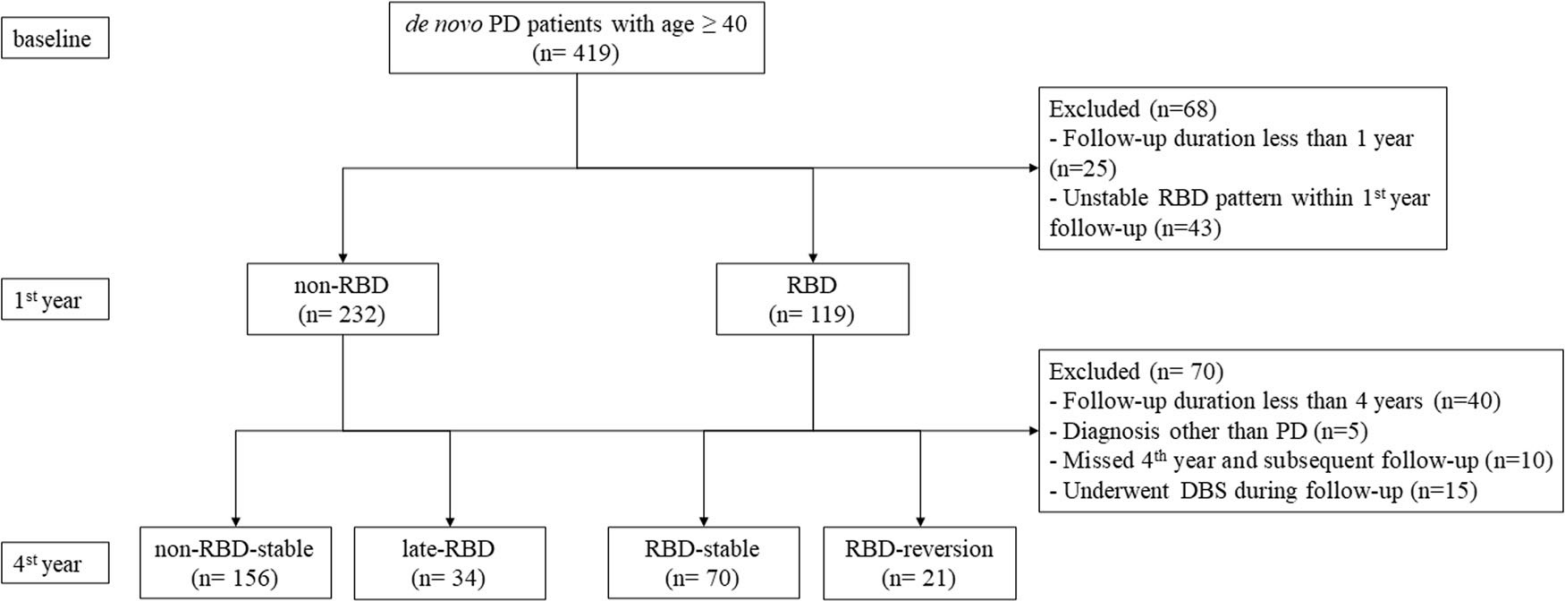
Flowchart of the study participants. DBS deep brain stimulation, PD Parkinson’s disease, RBD REM sleep behavior disorder.

A total of 281 de novo PD patients were included in this study and underwent a mean follow-up of 6.8 years. Among those participants, 91 (32.4%) had pRBD at baseline. As shown in Fig. 1, during the first 4 years of follow-up, RBD symptoms remained stable in 70 patients (24.9%, the RBD-stable group) but disappeared in 21 patients (7.5%, the RBD-reversion group), with a mean phenotype conversion time of 33.5 months. Additionally, 34 PD patients without pRBD at baseline developed RBD symptoms (12.1%, the late-RBD group), with a mean phenotype conversion time of 38 months, while 156 PD patients remained free of pRBD (55.5%, the non-RBD-stable group).

Demographics and clinical characteristics at baseline are summarized in Table 1. There was no significant difference in age, years of education, or follow-up duration among the four groups. There were slightly more males in the RBD-stable group compared with the non-RBD-stable group and the RBD-reversion group (*p* = 0.01 > 0.008, *p* = 0.012 > 0.008, respectively). Similarly, no significant difference was found in H&Y stage or MoCA score at baseline. RBD-stable patients showed higher MDS-UPDRS part III scores compared with non-RBD-stable and late-RBD patients at baseline (*p* = 0.004 < 0.008, *p* = 0.005 < 0.008, respectively). 2 patients with H&Y stage 3 or higher, 35 patients with MCI and 65 patients with MoCA < 26 were found at baseline, with no significant difference among different pRBD evolution patterns. The RBD-stable group showed lower HVLT-R recognition discrimination index scores than the non-RBD-stable group (*p* < 0.001 < 0.008).

**Table 1:** Patient demographics and clinical characteristics at baseline.

	Non-RBD-stable (*n* = 156)	Late-RBD (*n* = 34)	RBD-stable (*n* = 70)	RBD-reversion (*n* = 21)	*p* value
*Demographics*					
Age, y	61.8 ± 9.0	64.1 ± 8.8	63.0 ± 8.9	63.2 ± 10.5	0.467
Male, *n* (%)	98 (62.8%)	23 (67.6%)	56 (80.0%)	11 (52.4%)	**0.035**
years of education, y	15.8 ± 3.0	15.8 ± 3.2	15.6 ± 2.8	15.7 ± 3.1	0.854
follow-up duration, y	6.93 ± 1.0	6.93 ± 1.3	6.66 ± 1.6	6.28 ± 1.3	0.402
Time to RBD status change, y	/	3.16 ± 0.67	/	2.79 ± 0.96	/
*Motor function*					
MDS-UPDRS part III	19.3 ± 7.6	18.7 ± 8.7	23.1 ± 9.3	16.8 ± 9.1	**0.005**^a,b^
H&Y stage (stage 1/stage 2, *n*)	70/84	18/16	23/47	13/8	0.059
H&Y stage ≥3 at baseline, *n* (%)	2 (1.3%)	0	0	0	0.656
*Cognitive function*					
MoCA	27.5 ± 2.2	26.9 ± 2.3	26.8 ± 2.4	26.2 ± 3.0	0.071
MoCA <26 at baseline, *n* (%)	35 (22.4%)	7 (20.6%)	17 (24.3%)	6 (28.6%)	0.904
HVLT-R total recall *t*-score	46.04 ± 11.2	47.9 ± 11.5	44.2 ± 10.1	48.6 ± 11.0	0.251
HVLT-R Recognition Discrimination	46.9 ± 10.8	46.3 ± 10.8	41.6 ± 10.8	43.8 ± 11.5	**0.006**^a^
Index *t*-score					
JLO scaled score	12.5 ± 2.8	12.2 ± 2.7	11.8 ± 3.2	12.0 ± 2.8	0.446
LNS scaled score	11.8 ± 2.8	11.4 ± 2.6	11.0 ± 2.4	11.2 ± 3.3	0.332
SFT *t*-score	50.8 ± 9.8	51.1 ± 10.3	51.0 ± 9.2	49.5 ± 11.1	0.935
SDMT *t*-score	47.0 ± 8.8	44.2 ± 10.1	43.0 ± 10.1	43.7 ± 7.9	0.087
MCI at baseline, *n* (%)	14 (9.0%)	4 (11.8%)	15 (21.4%)	2 (9.5%)	0.069

*P* values <0.05 are highlighted in bold text. Pairwise comparisons with Bonferroni correction were performed. Significant *p* values are indicated with the following letters:

*HVLT-R* Hopkins verbal learning test revised, *H&Y* Hoehn–Yahr, *JLO* Judgment of Line Orientation, *LNS* Letter–Number Sequencing, *MCI* Mild Cognitive impairment, *MDS-UPDRS* Movement Disorder Society Unified Parkinson’s Disease Rating Scale, *MoCA* Montreal Cognitive Assessment, *SFT* Semantic Fluency Test, *SDMT* Symbol Digit Modalities Test.

^a^Comparison between the non-RBD-stable group and the RBD-stable group.

^b^Comparison between the late-RBD group and RBD-stable group.

### The relationship between pRBD evolution pattern and the progression of motor and cognitive impairment in PD

**Fig. 2:**
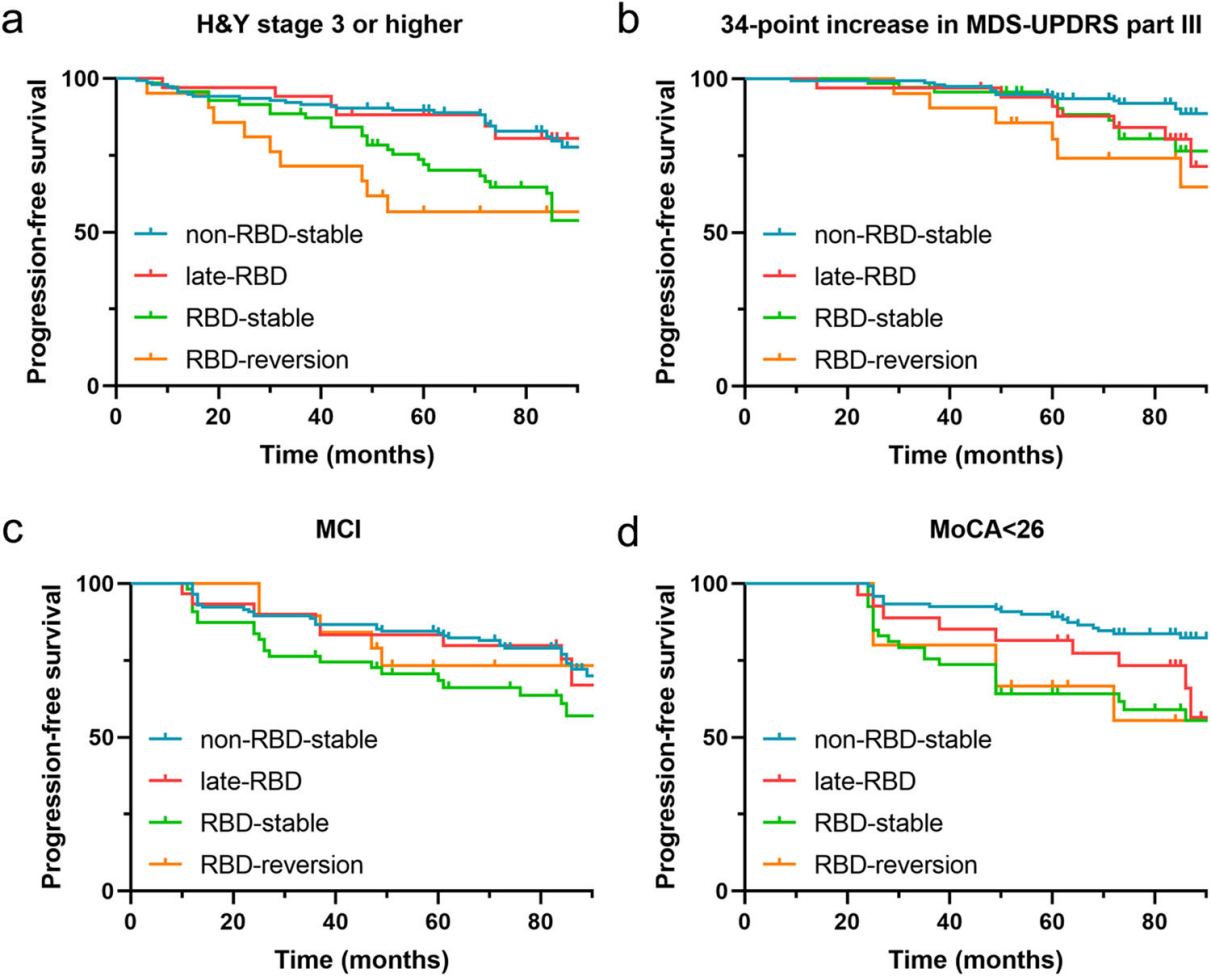
Kaplan–Meier survival curves for progression-free survival according to pRBD evolution pattern. Time from baseline to **a** Hoehn–Yahr stage ≥3, **b** 34-point increase in Movement Disorder Society Unified Parkinson’s Disease Rating Scale part III, **c** mild cognitive impairment according to MDS criteria (<1.5 SD on 2 or more tests), **d** Montreal Cognitive Assessment score <26 at 2 consecutive visits. Ticks indicate censoring events.

Kaplan–Meier analyses were performed to analyze whether different pRBD evolution patterns were associated with motor progression in PD. As shown in Fig. 2a, the RBD-reversion group exhibited a shorter progression-free survival (PFS) time than the non-RBD-stable group and the late-RBD group (69.2 months vs 95.9 months, *p* < 0.001; 69.2 months vs 95.3 months, *p* = 0.019; respectively) for progression to H&Y stage 3 or higher. The RBD-stable group also had a shorter PFS time, albeit to a lesser extent, compared with the non-RBD-stable group (82.4 months vs 95.9 months, *p* < 0.001). Regarding progression to a 34-point increase in MDS-UPDRS part III score (Fig. 2b), the RBD-reversion group showed a shorter PFS time than the non-RBD-stable group (83.2 months vs 102.4 months, *p* = 0.001). The late-RBD group also exhibited a slightly shorter PFS time than the non-RBD-stable group (95.2 months vs 102.4 months, *p* = 0.047). Cox proportional hazards analysis for each outcome was also performed. As shown in Table 2, we found that the RBD-reversion type was associated with motor symptom progression, including progression to H&Y stage 3 or higher (HR = 3.26, 95% CI 1.59–6.70, *p* = 0.001) and a 34-point increase in MDS-UPDRS part III scores (HR = 3.92, 95% CI 1.63–9.41, *p* = 0.002). The RBD-stable type tended to be associated with motor symptom progression, mainly in progression to H&Y stage 3 or higher (HR = 2.37, 95% CI 1.41–4.00, *p* = 0.001). The late-RBD type might have a mild impact on progression to 34-point increase in MDS-UPDRS part III scores. The year-by-year MDS-UPDRS part III score comparisons can be found in Supplementary Table 1. To decrease the effects of differences in years of follow-up, the other MDS-UPDRS cut-off score was used (years of follow-up multiplied by 5-point increase per year for each patient). As shown in Supplementary Fig. 1, Kaplan–Meier analysis revealed that the RBD-reversion group still showed a shorter PFS time than the non-RBD-stable group (81.9 months vs 101.0 months, *p* = 0.025). Cox proportional hazards analysis found that the RBD-reversion type was associated with increase in UPDRS part III scores (HR = 2.81, 95% CI 1.12–7.06, *p* = 0.028, Supplementary Table 2).

**Table 2:** Estimated HRs for motor progression in different pRBD evolution patterns.

Outcome	late-RBD	*p* value	RBD-stable	*p* value	RBD-reversion	*p* value
	HR (95% CI)		HR (95% CI)		HR (95% CI)	
Conversion to H&Y stage ≥3	1.09 (0.478–2.48)	0.840	**2.37 (1.41**–**4.00)**	**0.001**	**3.26 (1.59**–**6.70)**	**0.001**
MDS-UPDRS part III 34-point increase	2.22 (0.965–5.12)	0.060	1.66 (0.809–3.42)	0.167	**3.92 (1.63**–**9.41)**	**0.002**

Cox regression, compared with the non-RBD-stable group, adjusted for sex. Bonferroni correction was performed for multiple comparisons (α = 0.05/3 = 0.017). *P* values < α are highlighted in bold text.

*HR* hazard ratio, *H&Y* Hoehn–Yahr, *MDS-UPDRS* Movement Disorder Society Unified Parkinson’s Disease Rating Scale.

The RBD-stable type was found to be associated with fast progression to MCI, as Kaplan–Meier analysis revealed a shorter mean PFS time in that group than in the non-RBD-stable group (78.6 months vs 85.5 months, *p* = 0.034, Fig. 2c), while the RBD-reversion group exhibited a nonsignificant tendency towards a shorter mean PFS time. Regarding conversion to MoCA < 26, the RBD-stable group and the RBD-reversion group both showed shorter PFS time compared with the non-RBD-stable group (79.8 months, 73.4 months vs 98.4 months, *p* < 0.001, *p* = 0.009, respectively, Fig. 2d). Longitudinal domain-specific neuropsychological assessments were also analyzed in Supplementary Table 3. The RBD-stable type showed association with decline in verbal memory and executive function, while the RBD-reversion type was associated with decline in verbal memory. No significant difference was observed in other cognitive domains.

### Gray matter and cortical thickness alterations at baseline in PD patients with different pRBD evolution patterns

**Fig. 3:**
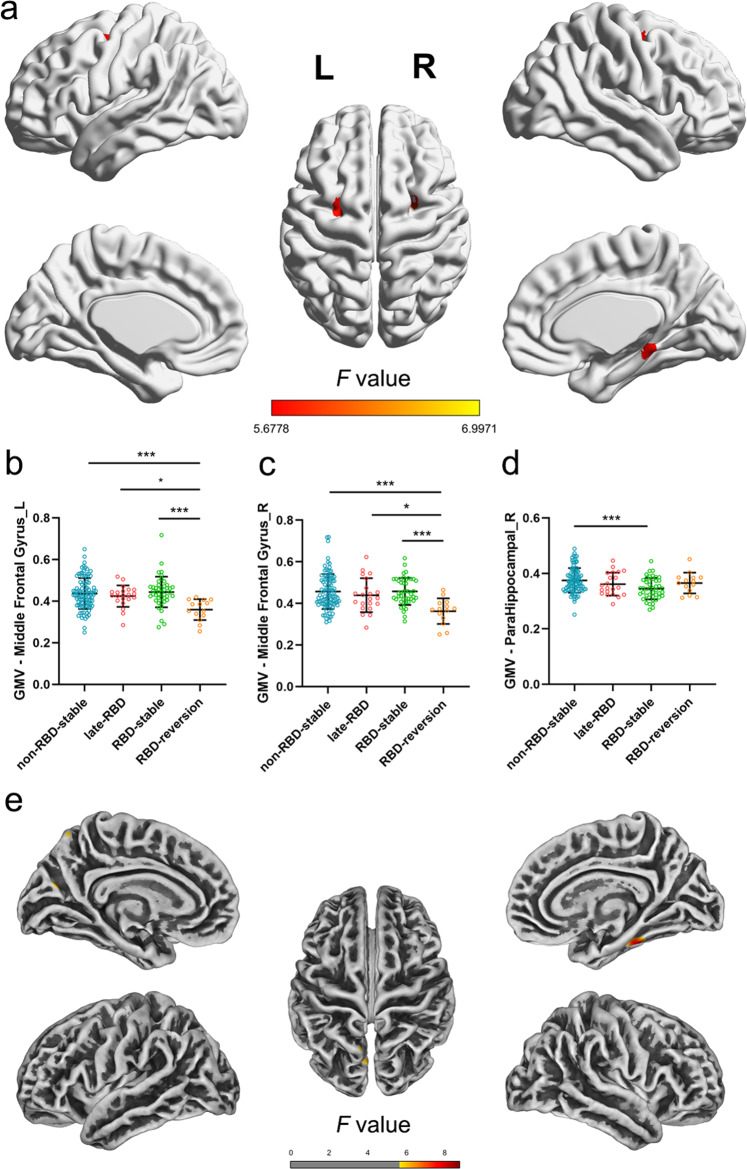
Gray matter volume and cortical thickness differences among pRBD evolution patterns. **a** Regions with gray matter volume differences among groups. *Post hoc* ROI analysis in **b** left middle frontal gyrus, **c** right middle frontal gyrus, **d** right para-hippocampus (*p < 0.05, **p < 0.01, ***p < 0.001, Bonferroni correction, error bars represent SD). **e** Regions with cortical thickness differences among groups.

We hypothesized that in PD patients with different pRBD evolution patterns, distinct lesions in brain regions associated with motor or cognitive function existed at baseline. Whole-brain VBM and SBM analyses were thus performed in 97 non-RBD-stable, 21 late-RBD, 45 RBD-stable, and 14 RBD-reversion patients. PD patients with different pRBD evolution patterns had different GM atrophy patterns (uncorrected *p* < 0.001, cluster size > 20 voxels, Fig. 3a), which were mainly located in the bilateral middle frontal gyrus and the right para-hippocampal gyrus (Supplementary Table 4). Additionally, ROI-based *post hoc* analyses were performed. The RBD-reversion group had reduced gray matter volume (GMV) in the left middle frontal gyrus (adjusted *p* < 0.001, *p* = 0.019, *p* < 0.001, compared with the non-RBD-stable group, the late-RBD group and the RBD-stable group respectively, Fig. 3b) and right middle frontal gyrus (adjusted *p* < 0.001, *p* = 0.033, *p* < 0.001, compared with the non-RBD-stable group, the late-RBD group and the RBD-stable group respectively, Fig. 3c). Moreover, the RBD-stable group had reduced GMV in the right para-hippocampal gyrus compared with the non-RBD-stable group (adjusted *p* < 0.001, Fig. 3d).

PD patients with different pRBD evolution patterns also had different cortical thickness alteration patterns (*p* < 0.001, uncorrected at peak level, Fig. 3e), which were mainly located in the right fusiform, the left cuneus and the left superior parietal lobe (Supplementary Table 5).

Longitudinal VBM analyses were further performed in 42 in non-RBD-stable patients, 6 late-RBD patients, 19 RBD-stable patients and 5 RBD-reversion patients at the 4th year follow-up. As shown in Supplementary Fig. 2, Group X time interaction analysis revealed significant gray mater atrophy in para-hippocampal gyrus during the disease progression between the non-RBD-stable group and the RBD-stable group.

## Discussion

In the present study, longitudinal changes in pRBD in de novo PD patients were studied. First, we confirmed that the course of pRBD in PD fluctuated over time, and could be classified into four types, labeled the non-RBD-stable, late-RBD, RBD-stable, and RBD-reversion patterns. Second, we identified a relationship between pRBD evolution patterns and the progression of motor and cognitive impairment in PD patients. Specifically, the non-RBD-stable type showed relatively mild disease progression, while the RBD-reversion type showed the fastest motor progression and the RBD-stable type showed the fastest cognitive decline. Third, we demonstrated that PD patients with different pRBD evolution patterns had dissimilar gray matter and cortical thickness alteration patterns at disease onset. Interestingly, relevant brain regions were associated with both REM sleep and motor/cognitive functions. These combined results suggested the potential prognostic value of pRBD evolution pattern in PD.

An altered overall prevalence of pRBD has been observed in previous longitudinal cohort studies, in which the majority reported an elevated overall RBD prevalence during follow-up^6,7,12^. In our study, up to the 4th year of follow-up, 12.1% of PD patients who were free of pRBD at baseline developed pRBD, while pRBD disappeared in 7.5% of PD-RBD patients. A net increase was found in the number of PD-RBD patients, which was consistent with previous literature. We reported a lower rate of RBD reversion than a previous work^9^. This might be because a stricter definition of RBD reversion (with RBDSQ scores < 5 at two consecutive visits) was adopted in this study. We did not calculate the proportion of answers given by the bed partners in RBDSQ, because PPMI did not record at individual level whether the specific patient had a bed partner. Future studies using questionnaires assessing pRBD may consider record if patients had bed partners and if the answer were given by the bed partners.

In previous cross-sectional^13^ and longitudinal^14^ studies, the rate of RWA has been reported to increase with PD progression. Interestingly, previous study also found that DBE disappeared in some PD patients at follow-up^15^. In the present study, we observed that pRBD fluctuated with the course of PD. It is possible that DEB may persist or disappear whereas RWA may continue to develop. This discrepancy may reflect the difference in the stage of neurodegenerative disorders. Thus, the evolution pattern of pRBD might have a prognostic value. Future studies using PSG can further explore the differences in the course of DEB and RWA and the underlying mechanisms.

This study focused on the association between the variation of pRBD course and PD progression. Previous studies demonstrated that the presence of RBD, especially at disease onset, is an independent predictor of faster deterioration of both motor and cognitive function^4,5,16^. A similar result was also found in MSA^17^. Here, we found that not only the presence of RBD but also the early variation of pRBD course was associated with distinct rates of motor and cognitive progression, which generally showed the following tendencies: RBD-reversion > RBD-stable > late-RBD > non-RBD-stable in terms of motor progression, while RBD-stable > RBD-reversion > late-RBD ≈ non-RBD-stable in terms of cognitive decline. The two types with the worst prognosis (RBD-reversion and RBD-stable) constituted the PD-RBD group at baseline. This observation was closely consistent with previous cross-sectional studies. Interestingly, the RBD-reversion type is more related to motor progression, while the RBD-stable type is more related to cognitive decline, indicating that different pathological lesions and underlying mechanisms might exist within baseline PD-RBD. Therefore, the pRBD evolution pattern might have a better prognostic value. It is noted that the RBD-stable group had a higher MDS-UPDRS part III score at baseline, though the RBD-reversion group had a higher HR for motor progression. Considering that balance difficulties weight heavily in the H&Y scale^18^, this finding might suggest a potential association between RBD-reversion and postural instability in PD.

Previous literatures found a correlation between GM concentration of right middle frontal gyrus and UPDRS part III score^19^. Also, brain metabolic abnormalities in the middle frontal gyrus were reported to be associated with freeze of gait and correlate with the UPDRS part III subscore in PD^20^. These might explain the rapid motor progression of RBD-reversion patients in our study.

Moreover, a previous longitudinal study found that cortical thinning in parietal area was a significant predictor for the development of Lewy body disorder in idiopathic RBD (iRBD)^21^. We also found that different pRBD evolution patterns had distinct cortical thinning pattern in the left superior parietal lobe. iRBD is considered to be the prodromal stage of multiple neurodegenerative diseases, especially PD^22^. This finding further supports our hypothesis.

In addition, the RBD-stable group showed GM atrophy in the para-hippocampal gyrus, which is generally considered to be associated with cognitive decline^23^. Regional brain metabolism in the para-hippocampal gyrus was also found to correlate with executive, memory, and visuospatial function in PD patients with cognitive impairment^24^. These neuroimage findings are in good agreement with previous literature and could explain the clinical characteristics of different pRBD evolution patterns.

Longitudinal VBM analyses were further performed and revealed significant gray mater atrophy in para-hippocampal gyrus during the disease progression. To note, para-hippocampal gyrus has been reported to be associated with cognitive function, and that the RBD-stable type is more related to cognitive decline. It is possible that different pRBD evolution patterns have different forms of brain lesions during disease progression, resulting in different disease outcome. Future studies with larger sample size can further validate the abovementioned findings.

There are several limitations of our study to note. First, RBD symptoms were assessed using a questionnaire. Future studies with polysomnography (PSG) proven RBD patients are required to validate the abovementioned findings. However, questionnaire-based evaluation is relatively time-saving and practical, especially in healthcare settings where PSG was not available. And it is still widely used in recent clinical researches^5,25^. It should also be noticed that RBDSQ does not include questions about the intensity and frequency of RBD symptoms. It may be acceptable to use it to define large improvement and occurrence of RBD symptoms, considering that it has a rather high sensitivity (0.96)^26^ and sleep behaviors in RBDSQ are restricted to be within 6 months of the study visit. However, slight improvement and worsening of symptoms can be underestimated. Future longitudinal studies may consider using other questionnaires such as RBDQ-HK^27^. Second, the sample size of the RBD-reversion group is relatively limited. This is especially the case for the MRI data, with only 14 patients in the RBD-reversion group. Future studies with larger cohorts can better analyze the underlying mechanisms. Finally, variations in medication and dosages might interfere with pRBD and PD symptoms, although our study exclusively focused on de novo patients to minimize the treatment effect at baseline.

In conclusion, our study demonstrated that the early pRBD evolution pattern in de novo PD can be classified into four types with different prognoses as well as brain lesion patterns and can therefore be considered a potential predictor of motor progression and cognitive decline in PD.

## Methods

### Study design and participants

The data used in this study were acquired from the Parkinson’s Progression Markers Initiative (PPMI) database (www.ppmi-info.org/data). PPMI is an ongoing international multicenter longitudinal cohort of de novo PD patients (newly diagnosed within two years, untreated). Details including the aims and methods of the PPMI study have been published previously^28^ and are available on the PPMI website.

Data were downloaded from the PPMI database in April 2020. For this study, we included PD patients who met both of the following criteria: (1) men or women aged ≥ 40 years at baseline and (2) had available clinical assessment data. After enrollment, participants were followed up at 3-month intervals in the first year, 6-month intervals in the 4 following years, and 12-month intervals in the subsequent years. Patients were excluded if they (1) were followed up for less than 4 years; (2) missed the 4th year and the subsequent follow-up; (3) underwent DBS surgery during follow-up; or (4) were diagnosed with neurodegenerative diseases other than idiopathic PD during follow-up, including multiple system atrophy (MSA), dementia with Lewy bodies (DLB) and essential tremor (ET).

### Ethical approval

The PPMI study is registered at ClinicalTrials.gov (NCT01141023). Each participating PPMI site received approval from an ethical standards committee on human experimentation before the start of the study. Written informed consent for the study was obtained from all participating individuals.

### pRBD assessment and classification of pRBD evolution patterns

The presence of pRBD was assessed by the RBD Screening Questionnaire (RBDSQ)^26^. The RBDSQ is a widely used tool to assess RBD symptoms and has been validated in several populations, demonstrating both high sensitivity and specificity^29,30^. An RBDSQ score of 5 was used as the cutoff value as previously described^5^. In the present study, all of the sleep behaviors mentioned in RBDSQ were within 6 months from the relevant visit. To increase the stability of our findings, we defined pRBD as RBDSQ scores ≥5 at two consecutive visits.

Baseline RBD status was measured within 1 year from baseline, and the evolution pattern of pRBD was measured at the 4th year of follow-up. Namely, PD patients were defined as (1) RBD, if they had RBDSQ scores ≥ 5 at baseline and at the subsequent visit; or (2) non-RBD, if they had RBDSQ scores < 5 at baseline and at the subsequent visit. Patients were excluded if they did not reach a stable RBD status within the 1st year of follow-up. At the 4th-year follow-up, patients were classified into four groups according to their symptom fluctuation over time: (1) non-RBD-stable: patients were free of pRBD at baseline and throughout the 4-year follow-up; (2) late-RBD: patients were free of pRBD at baseline but developed pRBD within the four-year follow-up; (3) RBD-stable: patients had pRBD at baseline, and the RBD symptoms persisted during the four-year follow-up; and (4) RBD-reversion: patients had pRBD at baseline, but the symptoms disappeared or improved during the four-year follow-up. Patients with milder RBD symptoms (RBDSQ score of 1–4) compared with baseline were included in this subgroup.

To illustrate whether RBD-reversion was medication-induced, use of RBD medications, including clonazepam and melatonin was summarized in Supplementary Table 6. None of the patients in RBD-reversion group started RBD medications within 2 years before pRBD disappeared or improved.

### Clinical assessments

Motor symptom severity was measured with the Movement Disorder Society Unified Parkinson’s Disease Rating Scale (MDS-UPDRS) part III and Hoehn-Yahr (H&Y) stage. If patients were receiving PD medications, the MDS-UPDRS part III was performed while they were on and off their medication (further details are available on the PPMI website). For patients who were examined only while on medication, an off-medication score was estimated as previously described^31^. Neuropsychological tests were performed to assess global and domain-specific cognitive status, including: the Montreal Cognitive Assessment (MoCA) for global cognition; the Hopkins Verbal Learning Test-Revised (HVLT-R) for verbal memory; the Judgment of Line Orientation (JLO) for visuospatial ability; the Letter–Number Sequencing (LNS) for working memory; the Semantic Fluency Test (SFT) animal category for verbal fluency; and the Symbol Digit Modalities Test (SDMT) for executive function. Performances on these assessments were transformed to *t*-scores or scaled scores as previously described^32^.

### Outcomes

Motor progression was defined using two criteria: (1) H&Y stage: motor progression was defined as conversion to H&Y stage 3 or higher as previously described^33,34^. (2) MDS-UPDRS part III score: An increase of 4.63 points on the MDS-UPDRS part III was considered to represent clinically meaningful worsening of motor symptoms^35^. Therefore, fast motor progression was defined as a 34-point increase in MDS-UPDRS part III score (off-medication) based on the mean 6.8 years of follow-up in this study (mean of 5 points per year).

Cognitive decline was defined using two criteria according to Movement Disorder Society Task Force Guidelines^36^: (1) mild cognitive impairment (MCI) was diagnosed if the patient scored at least ≤1.5 SD from the normative mean on 2 or more neuropsychological tests (the HVLT-R total recall and recognition discrimination index count as 1 test). (2) MoCA score: patients with a MoCA score of < 26 at 2 consecutive visits were considered to be MCI as previously described^5,37^.

Patients with H&Y stage ≥ 3, MCI, or MoCA < 26 at baseline were shown in Table 1 and excluded from corresponding survival analyses.

### Neuroimaging

203 patients had available high-resolution three-dimensional T1-weighted MRI scans that were performed within 6 months from baseline. 17 patients were excluded due to large discrepancies in the scan protocol; 6 patients were excluded due to significant abnormalities on MRI (e.g., infarct, neoplasm) and 3 patients were excluded due to excessive head-motion artifacts. T1-weighted images of 145 patients were acquired using the following parameters: matrix *x* = 256, *y* = 240–256, *z* = 160–192; repetition time (TR) = 1900–2300 ms; echo time (TE) = 2.3–3.2 ms; slice thickness = 1 mm; flip angle = 9–15. T1-weighted images of 13 patients were acquired using the following parameters: matrix *x* = 256, *y* = 256, *z* = 170; TR = 7–7.3 ms; TE = 3.2–3.4 ms; slice thickness = 1 mm; flip angle = 8. T1-weighted images of 19 patients were acquired using the following parameters: matrix *x* = 256–268, *y* = 255–256, *z* = 152–170; TR = 6.8–9.1 ms; TE = 3.2–3.6 ms; slice thickness = 1.2 mm; flip angle = 8–13. Further details can be found in the PPMI MRI operation manual.

Voxel-based morphometry (VBM) analysis and surface-based morphometry (SBM) analysis were performed as previously described^38–40^. Briefly, we used Statistical Parametric Mapping 12 (SPM12, Wellcome Department of Imaging Neuroscience, London, UK) and Computational Anatomy Toolbox 12 (CAT12, Gaser C, Jena University Hospital, http://www.neuro.uni-jena.de/cat/) software for imaging data analysis. T1-weighted images were segmented into gray matter (GM), white matter (WM), and cerebrospinal fluid (CSF) and spatially normalized. Cortical thickness was estimated using a projection-based methodology by calculating the distance between the inner (boundary between WM and GM) and outer (boundary between GM and CSF) cortical surfaces. Scans were smoothed by an 8 mm full-width at half-maximum (FWHM) isotropic Gaussian kernel for VBM analysis. All surface measures were resampled and smoothed with a Gaussian kernel of 15 mm (FWHM).

### Statistical analysis

Statistical analyses were performed using SPSS 25 (IBM Corp., Armonk, NY). Continuous data are presented as the mean ± SD and were analyzed with one-way analysis of variance (ANOVA) if normally distributed; otherwise, Kruskal–Wallis test was used. Bonferroni correction was applied for multiple comparisons. Categorical data are expressed as frequencies and percentages and were compared using chi-square tests or Fisher’s exact tests. The Kaplan–Meier method was used to analyze PFS, and the log-rank test was performed to compare survival rates among groups. Logistic regression was used to estimate odds ratios (OR) and Cox proportional hazards model was used to estimate hazard ratios (HR), which were adjusted for sex. A generalized linear mixed model was used to estimate the longitudinal sex-adjusted effect of pRBD evolution patterns on cognitive domain scores. For VBM analyses, age, sex, total intracranial volume (TIV), and imaging parameters were used as covariates. A voxel-wise threshold of *p* < 0.001, uncorrected for multiple comparisons, with a minimum cluster size of 20 voxels was considered significant^41^. The mean value of all voxels within the significant cluster was extracted for region of interest-based *post hoc* analyses. For SBM analyses, age, sex, and imaging parameters were used as covariates. Atlas labeling was performed according to Desikan–Killiany atlas^42^.

### Reporting summary

Further information on research design is available in the Nature Research Reporting Summary linked to this article.

## Supplementary information

Supplementary information

Reporting Summary

## Data Availability

All data reported in this article are available in the PPMI database (http://ppmi-info.org).
