# Supplementary material for: Evolution patterns of probable REM sleep behavior disorder predicts Parkinson’s disease progression
Source: NPJ Parkinsons Dis. 2022 Apr 5;8:36. doi: 10.1038/s41531-022-00303-0 (PMC8983711; doi:10.1038/s41531-022-00303-0)
Supplement: Supplementary file 1 — Supplementary information [file 41531_2022_303_MOESM1_ESM.pdf]

Supplementary Table 1. Longitudinal comparisons of MDS-UPDRS part III score among groups.

	non-RBD-stable	late-RBD	RBD-stable	RBD-reversion	*p* values
1st-year follow-up	24.1±9.7	23.8±11.3	27.6±11.6	23.8±10.4	0.202
2nd-year follow-up	27.2±11.0	27.4±14.2	31.3±11.2	29.0±10.6	0.080
3rd-year follow-up	28.3±11.5	29.0±13.7	33.9±11.5	30.6±14.2	**0.005**
4th-year follow-up	30.6±11.4	32.9±15.0	36.2±12.4	30.7±15.3	**0.018**
5th-year follow-up	30.4±11.5	32.5±14.5	38.7±14.0	36.6±18.1	**0.001**

P values <0.05 are highlighted in bold text.

Supplementary Table 2. Estimated HRs for motor progression in different pRBD evolution patterns using adjusted MDS-UPDRS cut-off

Outcome	late-RBD		RBD-stable		RBD-reversion	
	HR (95% CI)	*p* value	HR (95% CI)	*p* value	HR (95% CI)	*p* value
5-points increase per year in MDS-UPDRS part III (adjusted) [1]	2.04 (0.974-4.26)	0.059	1.52 (0.804-2.88)	0.197	**3.27 (1.47-7.31)**	**0.004**

Cox regression, compared with the non-RBD-stable group, adjusted for sex. Bonferroni correction was performed for multiple comparisons (α=0.05/3=0.017). *P* values < α are highlighted in bold text.

[1] For patients with follow-up time below average (< 6.8 years), the cut-off value was set as 5-points increase per year multiplied by follow-up years.

Abbreviations: HR, hazard ratio; MDS-UPDRS, Movement Disorder Society Unified Parkinson's Disease Rating Scale

Supplementary Table 3. Longitudinal associations of cognitive domain scores with RBD evolution patterns

Cognitive domain	late-RBD		RBD-stable		RBD-reversion	
	β	*p* value	β	*p* value	β	*p* value
Verbal memory	0.432	0.213	0.769	**0.004**	1.01	**0.011**
Visuospatial ability [1]	0.001	0.999	1.787	0.999	1.847	0.999
Working memory [1]	0.038	0.999	0.026	0.999	5.704	0.999
Verbal fluency [1]	-15.3	0.993	-15.5	0.992	-16.3	0.997
Executive function	0.666	0.262	1.38	**<0.001**	0.720	0.262

Generalized linear mixed model, compared with the non-RBD-stable group. Bonferroni correction was performed for multiple comparisons (α=0.05/3=0.017). P values < α are highlighted in bold text.

[1] model not significant.

Supplementary Table 4. Locations and MNI coordinates for the significant clusters in VBM analysis

Anatomical location	MNI coordinates			Number	Peak	p
	X	Y	Z	of voxels	*F*-value	(uncorrected)
Middle Frontal Gyrus_R	22.5	-3	54	36	6.99	<0.001
ParaHippocampal_R	13.5	-36	-10.5	24	6.73	<0.001
Middle Frontal Gyrus_L	-25.5	-4.5	51	38	6.68	<0.001

The X, Y, Z coordinates accord with the Montreal Neurological Institute (MNI) atlas.

Each location indicates the peak voxel with the highest statistical strength.

Supplementary Table 5. Locations for the significant clusters of cortical thickness in SBM analysis

Overlap of atlas region	Cluster size	Peak *F*-value	*P* (uncorrected)
100% right fusiform	247	8.0	<0.001
100% left cuneus	36	6.2	<0.001
100% left superior parietal lobe	29	6.0	<0.001

The Desikan-Killiany atlas was used to define regions of interest for the brain cortex.

Supplementary Table 6. Use of RBD medications at followups

	RBD-stable	RBD-reversion
Numbers of patients using RBD medications [1]		
throughout follow-up	26	5
within 4 years from baseline	21	3
before change of pRBD status [2]	/	0

[1] Medications include clonazepam and melatonin.

[2] RBD medications were given within 2 years before pRBD disappeared or improved.

Supplementary Figure 1.

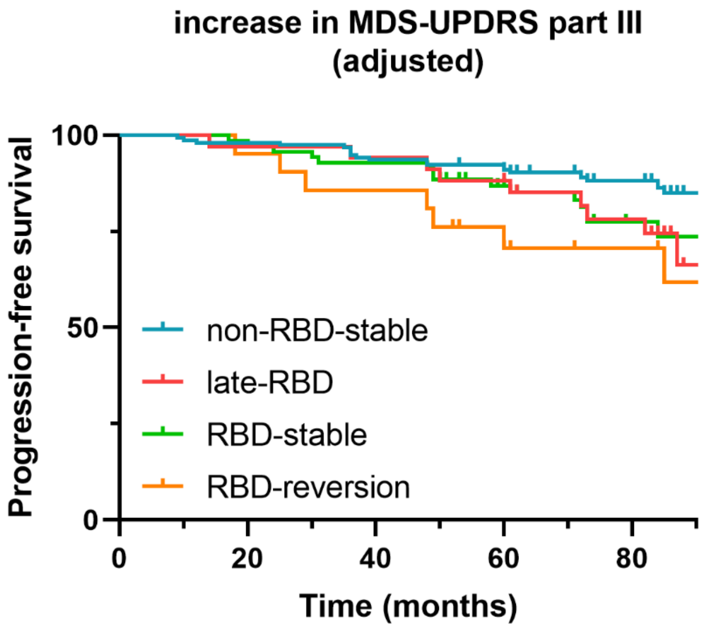

Time from baseline to increase in MDS-UPDRS part III (adjusted cut-off values: follow-up years multiplied by 5-points increase per year for patients with follow-up time below average).

Abbreviations: MDS-UPDRS, Movement Disorder Society Unified Parkinson's Disease Rating Scale

Supplementary Figure 2.

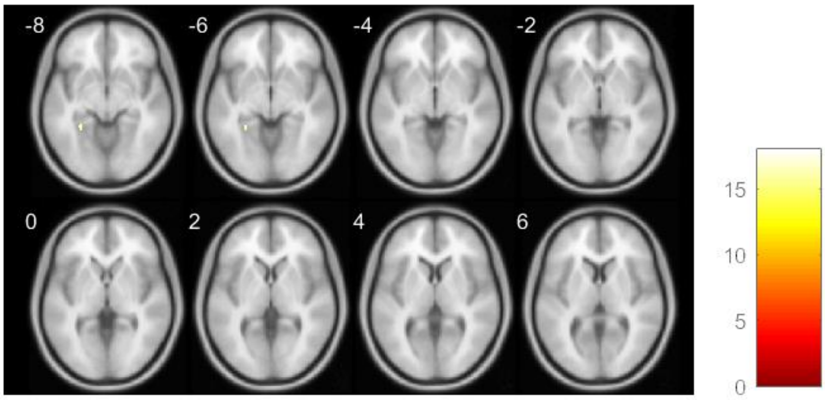

Regions with longitudinal differences in gray matter volume between the non-RBD-stable group and the RBD-stable group.
