# Supplementary material for: Evolution patterns of probable REM sleep behavior disorder predicts Parkinson’s disease progression
Source: NPJ Parkinsons Dis. 2022 Apr 5;8:36. doi: 10.1038/s41531-022-00303-0 (PMC8983711; doi:10.1038/s41531-022-00303-0)
Supplement: Supplementary file 2 — Reporting Summary [file 41531_2022_303_MOESM2_ESM.pdf]

nature portfolio

Corresponding author(s): Jun Liu

Last updated by author(s): Mar 2, 2022

Reporting Summary

Nature Portfolio wishes to improve the reproducibility of the work that we publish. This form provides structure for consistency and transparency in reporting. For further information on Nature Portfolio policies, see our Editorial Policies and the Editorial Policy Checklist.

Statistics

For all statistical analyses, confirm that the following items are present in the figure legend, table legend, main text, or Methods section.

n/a	Confirmed	
☐	☒	The exact sample size (*n*) for each experimental group/condition, given as a discrete number and unit of measurement
☐	☒	A statement on whether measurements were taken from distinct samples or whether the same sample was measured repeatedly
☐	☒	The statistical test(s) used AND whether they are one- or two-sided<br>*Only common tests should be described solely by name; describe more complex techniques in the Methods section.*
☐	☒	A description of all covariates tested
☐	☒	A description of any assumptions or corrections, such as tests of normality and adjustment for multiple comparisons
☐	☒	A full description of the statistical parameters including central tendency (e.g. means) or other basic estimates (e.g. regression coefficient) AND variation (e.g. standard deviation) or associated estimates of uncertainty (e.g. confidence intervals)
☐	☒	For null hypothesis testing, the test statistic (e.g. *F*, *t*, *r*) with confidence intervals, effect sizes, degrees of freedom and *P* value noted<br>*Give P values as exact values whenever suitable.*
☐	☒	For Bayesian analysis, information on the choice of priors and Markov chain Monte Carlo settings
☐	☒	For hierarchical and complex designs, identification of the appropriate level for tests and full reporting of outcomes
☐	☒	Estimates of effect sizes (e.g. Cohen's *d*, Pearson's *r*), indicating how they were calculated

Our web collection on statistics for biologists contains articles on many of the points above.

Software and code

Policy information about availability of computer code

Data collection	All data was collected using Office Excel 2013(Microsoft Corp.,Redmond, WA).
Data analysis	Statistical analysis was performed using SPSS 25 (IBM Corp., Armonk, NY).

For manuscripts utilizing custom algorithms or software that are central to the research but not yet described in published literature, software must be made available to editors and reviewers. We strongly encourage code deposition in a community repository (e.g. GitHub). See the Nature Portfolio guidelines for submitting code & software for further information.

Data

Policy information about availability of data

All manuscripts must include a data availability statement. This statement should provide the following information, where applicable:

- Accession codes, unique identifiers, or web links for publicly available datasets
- A description of any restrictions on data availability
- For clinical datasets or third party data, please ensure that the statement adheres to our policy

All data reported in this article are available in the PPMI database (http://ppmi-info.org).

Field-specific reporting

Please select the one below that is the best fit for your research. If you are not sure, read the appropriate sections before making your selection.

☒ Life sciences ☐ Behavioural & social sciences ☐ Ecological, evolutionary & environmental sciences

For a reference copy of the document with all sections, see nature.com/documents/nr-reporting-summary-flat.pdf

Life sciences study design

All studies must disclose on these points even when the disclosure is negative.

Sample size	No sample size calculation was performed. All cases meeting our inclusion criteria were included, and then those meeting exclusion criteria were exclude.
Data exclusions	68 cases were excluded according to pre-established exclusion criteria.
Replication	All data reported in this article are available in the PPMI database (http://ppmi-info.org). Patient selection were based on pre-established inclusion and exclusion criteria.
Randomization	This is a retrospective observational cohort study, so randomization is not relevant to the study.
Blinding	This is a retrospective observational cohort study, so blinding is not relevant to the study.

Reporting for specific materials, systems and methods

We require information from authors about some types of materials, experimental systems and methods used in many studies. Here, indicate whether each material, system or method listed is relevant to your study. If you are not sure if a list item applies to your research, read the appropriate section before selecting a response.

Materials & experimental systems

n/a	Involved in the study	
☒	☐	Antibodies
☒	☐	Eukaryotic cell lines
☒	☐	Palaeontology and archaeology
☒	☐	Animals and other organisms
☐	☒	Human research participants
☐	☒	Clinical data
☒	☐	Dual use research of concern

Methods

n/a	Involved in the study	
☒	☐	ChIP-seq
☒	☐	Flow cytometry
☐	☒	MRI-based neuroimaging

Human research participants

Policy information about studies involving human research participants

Population characteristics	At enrollment, PD subjects were required to be over 30 years old, untreated with PD medications , within 2 years of diagnosis. (Marek, K. et al. The Parkinson's progression markers initiative (PPMI) - establishing a PD biomarker cohort. Ann Clin Transl Neurol 5, 1460-1477, doi:10.1002/acn3.644 (2018).)
Recruitment	Enrollment was aided by a targeted recruitment program directed by MJFF(Michael J. Fox Foundation for Parkinson's Research) that provided study sites with customized recruitment strategies and materials. (Marek, K. et al. The Parkinson's progression markers initiative (PPMI) - establishing a PD biomarker cohort. Ann Clin Transl Neurol 5, 1460-1477, doi:10.1002/acn3.644 (2018).)
Ethics oversight	Each participating PPMI site received approval from an ethical standards committee on human experimentation before the start of the study. (Marek, K. et al. The Parkinson's progression markers initiative (PPMI) - establishing a PD biomarker cohort. Ann Clin Transl Neurol 5, 1460-1477, doi:10.1002/acn3.644 (2018).)

Note that full information on the approval of the study protocol must also be provided in the manuscript.

Clinical data

Policy information about clinical studies

All manuscripts should comply with the ICMJE guidelines for publication of clinical research and a completed CONSORT checklist must be included with all submissions.

Clinical trial registration	NCT01141023
Study protocol	Marek, K. et al. The Parkinson's progression markers initiative (PPMI) - establishing a PD biomarker cohort. Ann Clin Transl Neurol 5, 1460-1477, doi:10.1002/acn3.644 (2018).
Data collection	PD and HC subjects of similar age and gender from 24 study sites in the US, Europe and Australia from June 10, 2010 to July 17, 2020.
Outcomes	Mean Rates of Change including H&Y stage, MDS-UPDRS. Cognition tests including: the Montreal Cognitive Assessment (MoCA) for global cognition; the Hopkins Verbal Learning Test-Revised (HVLT-R) for verbal memory; the Judgment of Line Orientation (JLO) for visuospatial ability; the Letter–Number Sequencing (LNS) for working memory; the Semantic Fluency Test (SFT) animal category for verbal fluency; and the Symbol Digit Modalities Test (SDMT) for executive function.

Magnetic resonance imaging

Experimental design

Design type	T1-weighted MRI scans
Design specifications	Details can be found in the PPMI MRI operation manual
Behavioral performance measures	Details can be found in the PPMI MRI operation manual

Acquisition

Imaging type(s)	Structural.
Field strength	Details can be found in the PPMI MRI operation manual
Sequence & imaging parameters	Details can be found in the PPMI MRI operation manual
Area of acquisition	Details can be found in the PPMI MRI operation manual

Diffusion MRI ☐ Used ☒ Not used

Preprocessing

Preprocessing software	Computational Anatomy Toolbox - CAT12
Normalization	Data were normalized using Shooting Registration which includes linear and non-linear normalization. Details can be found in CAT12 manual (http://www.neuro.uni-jena.de/cat12/CAT12-Manual.pdf).
Normalization template	Geodesic Shooting templates in MNI space. These templates were derived from 555 healthy control subjects of the IXI-database (http://www.brain-development.org) and are available in the MNI space7 for six different iteration steps of Geodesic Shooting normalization. Details can be found in CAT12 manual (http://www.neuro.uni-jena.de/cat12/CAT12-Manual.pdf).
Noise and artifact removal	Patients with excessive head-motion artifacts were excluded before preprocessing.
Volume censoring	CAT12

Statistical modeling & inference

Model type and settings	multivariate - full factorial design
Effect(s) tested	ANOVA was used

Specify type of analysis: ☒ Whole brain ☐ ROI-based ☐ Both

Statistic type for inference (See Eklund et al. 2016)	voxel-wise
Correction	A voxel-wise threshold of p < 0.001, uncorrected for multiple comparisons, with a minimum cluster size of 20 voxels was considered significant

Models & analysis

n/a	Involved in the study
☒	☐ Functional and/or effective connectivity
☒	☐ Graph analysis
☐	☒ Multivariate modeling or predictive analysis

Multivariate modeling and predictive analysis	Age, sex, total intracranial volume (TIV) and imaging parameters were used as covariates.
